# Identification of a novel mutation in the *APTX* gene associated with ataxia-oculomotor apraxia

**DOI:** 10.1101/mcs.a002014

**Published:** 2017-11

**Authors:** Jingga Inlora, M. Reza Sailani, Hamidreza Khodadadi, Ahmad Teymurinezhad, Shinichi Takahashi, Jonathan A. Bernstein, Masoud Garshasbi, Michael P. Snyder

**Affiliations:** 1 Department of Genetics, Stanford University, Stanford, California 94305, USA;; 2 Department of Medical Genetics, School of Medicine, Shahid Beheshti University of Medical Sciences, Tehran 19839-63113, Iran;; 3 Department of Medical Genetics, School of Medicine, Ilam University of Medical Sciences, Ilam, Iran;; 4 Department of Pediatrics, Stanford University, Stanford, California 94305, USA;; 5 Department of Medical Genetics, Faculty of Medical Sciences, Tarbiat Modares University, Tehran, Iran;; 6 Department of Medical Genetics, DeNA Laboratory, Tehran, Iran

**Keywords:** apraxia, ataxia, athetosis, progressive cerebellar ataxia

## Abstract

Hereditary ataxias are a clinically and genetically heterogeneous family of disorders defined by the inability to control gait and muscle coordination. Given the nonspecific symptoms of many hereditary ataxias, precise diagnosis relies on molecular genetic testing. To this end, we conducted whole-exome sequencing (WES) on a large consanguineous Iranian family with hereditary ataxia and oculomotor apraxia. WES in five affected and six unaffected individuals resulted in the identification of a homozygous novel stop-gain mutation in the *APTX* gene (c.739A>T; p.Lys247*) that segregates with the phenotype. Mutations in the *APTX* (OMIM 606350) gene are associated with ataxia with oculomotor apraxia type 1 (OMIM 208920).

## INTRODUCTION

Hereditary ataxia is a group of disorders characterized by motor discoordination such as poor balance, abnormal eye and hand movements, and dysarthria (Bird 1993). In many cases, cerebral atrophy is also observed. To date, there are more than 30 autosomal dominant forms of ataxia and more than 60 forms that are either autosomal recessive or X-linked disease (Jayadev and Bird 2013). Many of the hereditary ataxias have overlapping presentations, and there is a high degree of genetic heterogeneity. For example, among the recessive forms of ataxia, the symptom of oculomotor apraxia is shared by oculomotor apraxia type 1 (AOA1), oculomotor apraxia type 2 (AOA2), oculomotor apraxia type 4 (AOA4), and ataxia telangiectasia (Bird 1993; Rothblum-Oviatt et al. 2016). Consequently, it can be difficult to devise an efficient strategy for targeted molecular testing in many cases. Using whole-exome sequencing (WES), we are able to identify a novel mutation in the *APTX* gene in a consanguineous Iranian family with hereditary ataxia. This mutation, c.739A>T, is predicted to result in a stop-gain mutation (p.Lys247*) in the aprataxin protein. *APTX* (OMIM 606350) is the only known gene to be associated with AOA1 (OMIM 208920). Characteristics of AOA1, such as motor disability, severe weakness, wasting, and discoordinated eye movements, are consistent with clinical features observed in the affected family members.

## RESULTS

### Clinical Presentation and Family History

**Figure 1.**
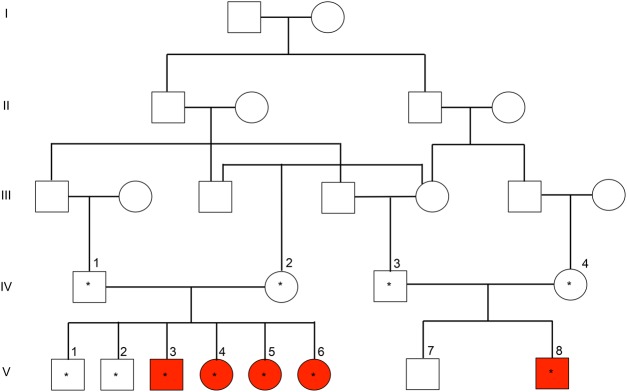
Pedigree of a consanguineous Iranian family with hereditary ataxia. Red boxes denote affected individuals. Boxes with asterisks indicate individuals who were analyzed by whole-exome sequencing.

The family pedigree is shown in Figure 1. This family is consanguineous and of Iranian ethnicity. Affected individuals, labeled in red, vary in age ranging from 8 (V-8) to 33 yr old (V-3) and show early onset of ataxia, ranging from the age 1 (V-8) to 6–7 yr old (V3-6). The clinical findings are summarized in Table 1. All affected family members show progressive ataxia and weak or loss of deep tendon reflex (DTR). These individuals are also dysarthric with hand athetosis and gaze palsy. There was no report on the presence of oculomotor ataxia. The parents of affected individuals (IV-1, IV-2, IV-3, and IV-4) are asymptomatic, which, along with family structure, suggests autosomal recessive inheritance.

**Table 1.** Iranian family member disease status and clinical descriptions

Family member	IV-1	IV-2	IV-3	IV-4	V-1	V-2	V-3	V-4	V-5	V-6	V-7	V-8
Age	64	55	41	41	40	35	33	30	27	24	7	5
Sex	Male	Female	Male	Female	Male	Male	Male	Female	Female	Female	Male	Male
Disease status	Healthy	Healthy	Healthy	Healthy	Healthy	Healthy	Affected	Affected	Affected	Affected	Healthy	Affected
Creatinine (mg/dl)	NA	NA	NA	NA	NA	NA	1.01 (Normal level)	0.87 (Normal level)	NA	0.66 (Normal level)	NA	NA
Cholesterol (mg/dl)	NA	NA	NA	NA	NA	NA	117 (Normal level)	148 (Normal level)	NA	173 (Normal level)	NA	NA
Albumin (g/dl)	NA	NA	NA	NA	NA	NA	3.6 (Normal level)	2.8 (Low)	NA	3.5 (Normal level)	NA	NA
Presence of oculomotor apraxia	NA	NA	NA	NA	NA	NA	NA	NA	NA	NA	NA	NA
Childhood onset (HP:0011463)	–	–	–	–	–	–	–	–	–	–	–	+; age 1
Juvenile onset (HP:0003621)	–	–	–	–	–	–	+; age 6–7	+; age 6–7	+; age 6–7	+; age 6–7	–	–
Ataxia (HP:0001251)	–	–	–	–	–	–	+	+	+	+	–	+
Weak deep tendon reflex (DTR) (HP:0001315)	–	–	–	–	–	–	+	+	+	+	–	+
Dysarthria (HP:0001260)	–	–	–	–	–	–	+	+	+	+	–	+
Hand athetosis (HP:0002305)	–	–	–	–	–	–	+	+	+	+	–	+
Gaze palsy (HP:0000605)	–	–	–	–	–	–	+	+	+	+	–	+

+, Family member exhibits this trait; –, family member does not exhibit this trait.

### Exome Sequencing

Because of the overlapping features of hereditary ataxia and the large number of genes (more than 400) associated with the disease, sequencing of ataxia-associated gene panels was not performed in this family. Thus, to pursue a comprehensive molecular genetic diagnosis, we performed WES on five affected and six unaffected family members (Fig. 1, individuals with an asterisk). Samples were sequenced with a mean depth of coverage of >80× (Table 2; Supplemental Fig. S1). Variant filtering and annotations were performed using VarSeq software (GoldenHelix Inc.). We identified 87,882 variants shared in all sequenced family members (Table 3). Variants were filtered based on (i) genotype quality (GQ) score of >20, (ii) read depth (DP) of >10, (iii) predicted loss-of-function or missense mutation, and (iv) minor allele frequency (MAF) of <0.01. MAFs were obtained from public databases including the Database for Short Genetic Variations (dbSNP) Common 144 (National Center for Biotechnology Information [NCBI]; http://ncbi.nlm.nih.gov/SNP/), 1000 Genome Project phase 3 (www.1000genomes.org), Exome Aggregation Consortium version 0.3 (EXaC; http://exac.broadinstitute.org/), National Heart, Lung, and Blood Institute (NHLBI) GO Exome Sequencing Project (Exome Variant Server, NLHBI Exome Sequencing Project [ESP)]; http://evs.gs.washington.edu/EVS/), and UK10K project (Avon Longitudinal Study of Parents and Children (ALSPAC)—Variant Frequencies 2013-11-01, GHI; https://www.uk10k.org/).

**Table 2.** Exome-sequencing coverage

Sample	Total sequences	Average read length (bp)	Percentage aligned reads (%)	Average on-target read coverage
IV-1	107,902,766	101	99.56	158.7
IV-2	119,110,694	101	99.65	173.9
IV-3	104,417,472	101	99.46	155.0
IV-4	118,306,342	101	99.70	176.8
V-1	108,913,590	101	99.51	160.0
V-3	122,629,182	101	99.53	177.0
V-4	94,647,342	101	99.59	135.0
V-5	114,642,644	101	99.43	168.1
V-6	123,450,772	101	99.65	182.0
V-7	121,395,560	101	99.67	180.0
V-8	88,617,944	101	99.38	131.2

**Table 3.** Variant filtering steps

Family member	IV-1	IV-2	IV-3	IV-4	V-1	V-3	V-4	V-5	V-6	V-7	V-8
Total variants	246,619	258,704	237,096	246,080	245,318	276,779	238,190	264,546	267,455	258,174	230,905
Shared variants	87,882										
GQ > 20 and DP > 10	56,092										
Effect (loss-of-function or missense)	34,675										
Homozygote variants	26 (Homozygote in affected members, but either heterozygote or homozygote for reference allele in controls)										
1KG MAF < 0.01	1										
EXaC MAF < 0.01	1										
dbSNP 144 MAF < 0.01	1										
NHLBI MAF < 0.01	1										
UK10K MAF < 0.01	1										
Exonic variants	1										
Candidate	Chr9:32984702; *APTX*; NM_001195248.1:c.739A>T; NP_001182177.1:p.Lys247*										

GQ, genotype quality; DP, read depth; MAF, minor allele frequency; 1KG, 1000 Genome Project phase 3; EXaC, Exome Aggregation Consortium; dbSNP, Database for Short Genetic Variations; NHLBI, National Heart, Lung, and Blood Institute; UK10K, UK 10000 project.

**Figure 2.**
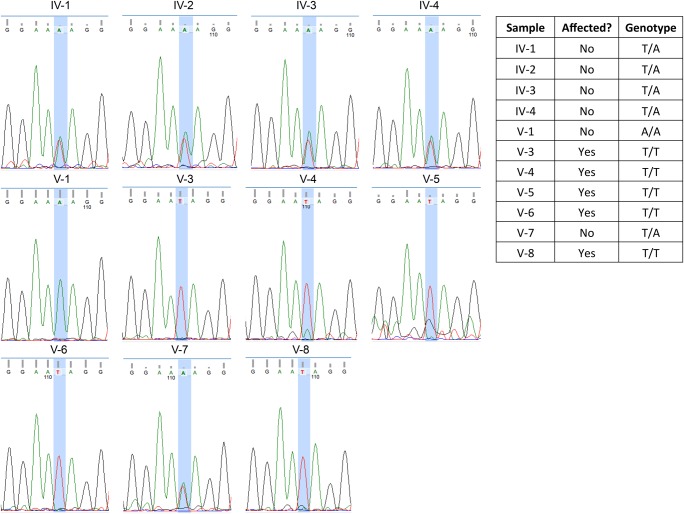
Sanger sequencing of family members reveal that the variant c.739A>T mutation segregates within the family among the affected and healthy controls. Note that Sanger sequencing was performed using a reverse primer. The genotype for affected individuals is T/T, whereas for healthy individuals it is A/A or T/A.

We filtered for variants that are found in the homozygous state in all five affected individuals but not in the healthy controls. Only one single-nucleotide polymorphism (SNP) passed this filtering step. *APTX* c.739A>T, located at Chr9:32,984,702, has no designated rsID (reference SNP identification number) and is expected to cause stop-gain mutation in APTX (p.Lys247*) (Table 4). This mutation occurs in exon 6 within the histidine triad (HIT) domain region, and may cause a deletion of the entire zinc finger domain of the protein. We further confirmed the presence of this variant by Sanger sequencing and determined that the variant segregates with disease within the family (Fig. 2).

**Table 4.** Summary of the variant reported in this study

Gene	Chromosome	HGVS coding DNA reference	HGVS protein reference	Predicted effect	Variant type	dbSNP ID if available	Genotype	ClinVar submission accession	ExAC highest MAF	Inheritance
*APTX*	Chr9:32984702	NM_001195248.1:c.739A>T	NM_001195248.1(APTX_i001): p.(Lys247*)	Lys247*	Stop- gained	NA	Homozygous	SCV000576420	NA	Homozygous recessive

HGVS, Human Genome Variation Society; dbSNP, Database for Short Genetic Variations; ExAC, Exome Aggregation Consortium; MAF, minor allele frequency; NA, not available.

## DISCUSSION

Using WES in a large consanguineous family, we identified a novel homozygous stop-gain mutation (c.739A>T) in the *APTX* gene, leading to a diagnosis of ataxia with AOA1.

In efforts to identify a cost-effective strategy to perform WES and analysis, we analyzed the sequencing reads by using the father–mother–proband (IV-1, IV-2, and IV-3, respectively) trio method. Using the same variant filtering strategy as in Table 3, we identified 54 potential gene candidates. Of these, two genes, including *APTX*, were previously reported to be associated with hereditary ataxia (Supplemental Table 1; Bird 1993; Jayadev and Bird 2013). We further identified the minimal number of samples required to yield similar results as analyzing 11 exomes. By reducing the sample size by more than half (i.e., analyzing only five individuals—three affected and two unaffected parents), we are able to narrow down our candidate gene to just *APTX* as previously shown (Supplemental Table 1).

Initial studies of AOA1 in Japanese and Portuguese populations identified linkage to a locus on Chromosome 9p13. Subsequent study determined that pathogenic mutations were within *APTX* (Date et al. 2001; Moreira et al. 2001b). A Portuguese study estimates that the prevalence of AOA1 to be 0.41 in 100,000 individuals (Barbot et al. 2001). APTX is a ubiquitous nuclear protein that is involved in single-stranded DNA break repair pathway (Moreira et al. 2001b; Mosesso et al. 2005). In the nervous system, APTX is also expressed in cerebellum, cerebral cortex, and spinal cord (Coutinho and Barbot 1993). APTX contains three main domains, namely a forkhead-associated (FHA) domain, a HIT domain, and a carboxy-terminal zinc finger domain (Seidle et al. 2005). Numerous pathogenic variants of *APTX* have since been identified and, interestingly, almost all variants (missense and truncating mutations) are found in the regions that encode the HIT domain (Date et al. 2001; Moreira et al. 2001a; Shimazaki et al. 2002; Le Ber et al. 2003; Sekijima et al. 2003; Tranchant et al. 2003; Amouri et al. 2004; Criscuolo et al. 2005; Castellotti et al. 2011; Rana et al. 2013). An in vitro study demonstrates that disease-associated mutations of APTX destabilize the protein and reduce its hydrolytic activity (Seidle et al. 2005; Hirano et al. 2007). This suggests that the HIT domain is important for the protein's stability and function.

In single-stranded DNA break repair (SSBR) pathway, binding of APTX to DNA catalyzes a nucleophilic release of the 5′ adenylate group from the single-stranded breaks, thereby restoring the ligatable 5′ phosphate terminus (Seidle et al. 2005). A mutated APTX in terminally differentiated cells such as neurons might be detrimental as it may block transcriptional activities thereby causing cell death. Indeed, fibroblasts from patients with AOA1 are more hypersensitive to oxidative damage than normal fibroblasts and that an increase in oxidative DNA damage was found in the cerebellum of the AOA1 patient (Harris et al. 2009).

Our exome-sequencing analysis reports a novel homozygous truncating mutation in *APTX* (c.739A>T; p.Lys247*). A previously identified truncating mutation, W279X, is also found on exon 6 of the *APTX* gene and occurs in the HIT domain of the protein (Moreira et al. 2001b; Tranchant et al. 2003; Habeck et al. 2004). Both in vitro studies and western blot analysis of patient samples have demonstrated that W279X mutation destabilizes the APTX protein (Seidle et al. 2005; Castellotti et al. 2011). Thus, together with previous studies, our results further support the importance of the catalytic activity of the protein in SSBR (Hirano et al. 2007). This study therefore expands the spectrum of pathogenic APTX mutations associated with AOA1.

## MATERIALS AND METHODS

### Exome Sequencing and SNP Calling

Library preparation was performed using a KAPA HyperPlus kit followed by exome capture using the IDT xGen Exome Research Panel v1.0. Briefly, 0.4 µg of gDNA was fragmented to a peak size of 150–200 bp using the KAPA Frag enzyme. The fragmented genomic DNA was end-repaired, A-tailed, ligated to indexing-specific paired-end adapters, and polymerase chain reaction (PCR)-amplified according to manufacturer's instructions. PCRs were cleaned using the Agencourt AMPure XP beads. To capture exonic regions, 500 ng of pooled libraries (eight libraries per pool) was hybridized to biotinylated oligonucleotides for 4 h at 65°C. The captured libraries were pulled down using Dynabeads MyOne Streptavidin M-270 (Invitrogen). A postcapture PCR was then performed to amplify the captured libraries and to add the barcode sequences for multiplex sequencing for 12 cycles. Afterward, amplified libraries were purified with AmpPure XP Beads. Qubit fluorometer and Bioanalyzer high-sensitivity chips were used to determine the final concentration of each captured library. The pooled libraries were paired-end-sequenced on a single Illumina HiSeq4000 lane at the Stanford Center for Genomics and Personalized Medicine according to standard protocols.

### Bioinformatics Analyses

DNA libraries were processed and analyzed using Sentieon whole-exome analysis workflow (Version 201611.01) with default settings (Sentieon Inc.; www.sentieon.com). Briefly, libraries were mapped with Burrows–Wheeler alignment (BWA)-mem software (Li and Durbin 2009) (version 0.7.12) to human genome (hg19 version), and then realigned around indels with GATK IndelRealigner. Next, base recalibration was performed with GATK BaseRecalibrator taking into account the read group, quality scores, and cycle and context covariates. Variants were called with GATK HaplotypeCaller to generate genome variant call format (gVCF). Variant filtering and annotation was done using Golden Helix VarSeq Version 1.1 software (Golden Helix Inc.; http://goldenhelix.com/products/VarSeq/). The gVCF of each sequenced family member is uploaded to VarSeq and organized by pedigree. Variants are filtered for GQ > 20 and DP > 10. Of the variants that pass the filter, only those that are predicted to cause loss-of-function or missense mutations are analyzed. Only variants with allele frequencies of <0.01 or missing were analyzed. The public databases that were used in this study for determining allele frequencies are dbSNP Common 144 (NCBI; http://ncbi.nlm.nih.gov/SNP/), 1000 Genome Project phase 3 (www.1000genomes.org), Exome Aggregation Consortium version 0.3 (EXaC; http://exac.broadinstitute.org/), NHLBI GO Exome Sequencing Project (Exome Variant Server, NLHBI Exome Sequencing Project [ESP]; http://evs.gs.washington.edu/EVS/), and UK10K project (ALSPAC—Variant Frequencies 2013-11-01, GHI; https://www.uk10k.org/). Based on the pedigree we predicted that the disease would follow an autosomal recessive pattern. Thus, we analyzed variants that are homozygous only in affected individuals.

The total number of reads, percentage of aligned reads, and average read lengths of each WES sample are calculated using SAMtools with stats option. To evaluate the efficiency of the exome capture and the total coverage along the targeted regions, BEDTools software (Broad Institute; Quinlan and Hall 2010) was used for the analysis. Briefly, BEDTools coverage with the –hist option was used to obtain a histogram coverage of each feature in the BED file of the Integrated DNA Technologies (IDT)’s exome probe target regions from the aligned bam files. The cumulative coverage plot of the exome-sequencing results was performed using R (the R Project for Statistical Computing; http://www.R-project.org/). Briefly, the total number of reads for each alignment was calculated from the summary histogram for all of the features in the BED file. This value is then used to calculate cumulative coverage (expressed as percentage of total read) for each coverage depth. A graph of capture target bases (%) versus depth is then plotted using R's plot function.

### Sanger Sequencing

To validate the candidate variant and to verify its segregation in the family, we used 5′-TGGACCCAGCAAAATCTACA-3′ as the forward primer and 5′-CAGGTGGTGGTGATAAAGGA-3′ as the reverse primer for PCR amplification of the variant sequence. PCR amplification was performed using following reagents: 25 µl REDTaq ReadyMix PCR Reaction Mix (Sigma-Aldrich), 1 µl forward primer (10 µM), 1 µl reverse primer (10 µM), 1 µl DNA (50 ng/µl), and 22 µl of water per PCR reaction. An initial denaturation step for 3 min at 94° was followed by 35 cycles of 30 sec at 94°, 30 sec at 57°, 30 sec at 72°, and the process completed by a final extension of 7 min at 72°. The PCR amplification resulted in a single DNA band on a standard 1% Agarose Gel, and was purified using Agencourt AMPure XP beads (Beckman Coulter, Inc.) before submitting for Sanger sequencing. The reverse primer 5′-CAGGTGGTGGTGATAAAGGA-3′ was used as sequencing primer. Samples were sent to Sequetech Inc. for Sanger sequencing using ABI 3130xl Genetic Analyzer.

## ADDITIONAL INFORMATION

### Data Deposition and Access

The family consented to the genetic study and publication of the genetic and clinical results. The variant described in this study has been submitted to ClinVar (http://ncbi.nlm.nih.gov/clinvar/) under accession number SCV000576420 (see Table 4). The exome-sequencing data has been deposited in the National Center for Biotechnology Information (NCBI) Sequence Read Archive (SRA) (http://www.ncbi.nlm.nih.gov/sra/) under SRA Study SRP106899.

### Ethics Statement

All participants, or their legal guardian, provided written and informed consent. The institutional review boards of Tarbiat Modares University and Stanford University reviewed the project. All the affected individuals underwent examination at the Medical Genetics Department, DeNA laboratory, Tehran.

### Acknowledgments

This study was supported by the National Institutes of Health (NIH) grants to M.P.S. (1P50HG00773501 and 8U54DK10255602). M.R.S is supported by grants from the Swiss National Science Foundation (SNSF) (P300PA_161005, P2GEP3_151825). We thank the Stanford Center for Genomics and Personalized Medicine for their sequencing services.

### Competing Interest Statement

M.P.S. is a cofounder of Personalis and a member of the scientific advisory boards of Personalis and Genapsys.

### Referees

Gholson Lyon

Anonymous

## Supplementary Material

Supplemental Material
